# Mindfulness-based online intervention increases well-being and decreases stress after Covid-19 lockdown

**DOI:** 10.1038/s41598-022-10361-2

**Published:** 2022-04-20

**Authors:** Francesco Bossi, Francesca Zaninotto, Sonia D’Arcangelo, Nicola Lattanzi, Andrea P. Malizia, Emiliano Ricciardi

**Affiliations:** 1 MoMiLab Research Unit, IMT School for Advanced Studies Lucca, Lucca, Italy; 2 Department of Psychology, School of Social and Behavioural Sciences, Kingston University, London, UK; 3 Intesa Sanpaolo Innovation Center SpA Neuroscience Lab, Turin, Italy; 4 Axes Research Unit, IMT School for Advanced Studies Lucca, Lucca, Italy

**Keywords:** Psychology, Human behaviour

## Abstract

Mindfulness interventions were shown to be effective in improving well-being and reducing perceived stress in several conditions. These effects were also found in online mindfulness-based training, especially in employees in organizational environments. The aim of this study was to test the effectiveness of an online mindfulness intervention on healthy employees, especially after the first Italian Covid-19 lockdown. Participants in the intervention group underwent an 8-week mindfulness online training program based on the Mindfulness-Based Stress Reduction (MBSR) protocol compared to a control (no-intervention) group. All participants filled in weekly surveys for the whole intervention duration via online questionnaires to measure their habits, mindfulness (FFMQ-15), emotion regulation (ERQ), positive and negative affect (PANAS), depression, anxiety and stress (DASS-21), resilience (RSA) and insomnia (ISI). 69 participants in the intervention group and 63 in the no-treatment control group were considered in the longitudinal analyses. We found significant differences between the intervention and control groups over time in the measures of mindfulness (in particular the nonreactivity subscale), positive affect, depression, and insomnia. Moreover, we found that the frequency of practice and ease perceived in practicing were positively correlated to several indices of well-being (mindfulness, positive affect, cognitive reappraisal) and negatively correlated to several indices of stress (negative affect, depression, anxiety, stress, insomnia, expressive suppression). These results show the importance and effectiveness of online mindfulness training programs to cope with stress among employees, especially after the Covid-19 lockdown.

## Introduction

### What is mindfulness

Mindfulness is an intrinsic and modifiable capacity of the human mind, commonly defined as “the awareness that emerges through paying attention on purpose, in the present moment, and nonjudgmentally to the unfolding of experience moment by moment”^1^. Mindfulness meditation, in turn, represents a systematic framework and process for cultivating mindfulness in daily life by intentional and sustained practice^2^.

The first mindfulness meditation program to be standardized in the 1970s by Jon Kabat-Zinn was the Mindfulness-Based Stress Reduction (MBSR) program^1^. This protocol consists of eight weekly sessions aimed at presenting and teaching different practices^3^. The goal of this program is to reduce perceived stress and to realize benefits for health and well-being. MBSR, first developed and standardized for patients with chronic pain^4–6^, demonstrated the benefits of mindfulness-based interventions (MBIs, broadly defined as any mindfulness-focused training protocol^7^) at both mental and body levels in helping people cope with many conditions^8^.

In the last decades, interest in research investigating mindfulness-based interventions has increased substantially. Khoury and colleagues^9,10^ showed that the MBSR program can provide a significant nonspecific moderate to large effect on reducing stress and increasing well-being in both healthy individuals and patients. In particular, there is an increasing body of evidence showing the clear effectiveness of mindfulness-based interventions in reducing stress, depression, and anxiety^7,11–13^.

When considering the psychological effects of mindfulness-based interventions, it is also crucial to consider the interaction between mindfulness and emotion regulation. Several studies found an overlap between them: awareness and acceptance, two components of mindfulness, are typically exploited in some emotion regulation strategies, both from a conceptual^14^ and from a neuroanatomical point of view^15^, even though interventions based on the two constructs differ fundamentally in terms of the underlying processes they address^16^. Concerning specific emotion regulation strategies, some studies found mindfulness-based interventions to be linked to increasing use of cognitive reappraisal and decreasing use of expressive suppression^17,18^. On the one hand, cognitive reappraisal is an advanced form of cognitive change that involves representing a potentially emotion-eliciting situation in a way that changes its emotional impact^19^ and it is related to experiencing and expressing greater positive emotion and lesser negative emotion (*ibidem*). On the other hand, expressive suppression is a basic form of response modulation that involves inhibiting ongoing emotion-expressive behaviour and it is linked to experiencing and expressing lesser positive emotion and greater negative emotion (*ibidem*). Therefore, previous literature suggests that mindfulness-based interventions foster more advanced and effective emotion regulation strategies. Moreover, several studies showed that mindfulness-based interventions improve the quality of sleep (typical index of well-being) and reduce the incidence of insomnia and sleep disorders^20–22^.

### Online mindfulness training programs and Covid-19 pandemic

When considering the exponential development of technology and the extensive availability of internet access, the overwhelming increase of online mindfulness interventions and apps over the last years is incontrovertible^23^. Indeed, digital mindfulness interventions offer several advantages, such as increased accessibility, anonymity, standardization, personalization and higher efficacy. Nevertheless, using mindfulness practices via online protocols also has many disadvantages to be kept into account: the possibility of low engagement, shallow learning, unaddressed obstacles and frustration. The presence of a trainer presenting the practices and that may be contacted for doubts or questions may help to solve these disadvantages. As a matter of fact, an extensive meta-analysis^24^ demonstrated significantly larger effect sizes for guided online mindfulness-based interventions compared to unguided ones. The same meta-analysis showed a consistent small to moderate beneficial effect of online mindfulness-based interventions on stress, depression, anxiety, well-being, and mindfulness.

Considering the ease of use, the number of smartphone apps focused on mindfulness practices (e.g., HeadSpace, Calm) has notably spread during the last years^25^. Therefore, smartphone apps may be a great instrument to familiarize with and increase the frequency of mindfulness practices. A systematic meta-analysis (*ibidem*) found a significant increase in mindfulness and lower levels of psychological stress in participants using mindfulness apps. Significant effects of these apps on mindfulness, well-being and perceived stress were found in the general population^26,27^, in healthy employees^28^ and categories prone to burnout, i.e., physicians^29^, or with high levels of stress, i.e., college students^30^, in particular, medical students^31^ and pharmacy students^32^.

Mindfulness-based interventions have been spreading among employees in the workplace during the latest years, proving their efficacy on emotional exhaustion and personal accomplishment (two dimensions of burnout), psychological distress, depression, anxiety, and occupational stress, as well as mindfulness, quality of sleep and relaxation^3^. The increase of online mindfulness training programs through video calls or apps has been exploited also in the workplace. Not only it proved effects on well-being comparable with internet-based cognitive-behavioural training^33^, but also on organizational parameters such as decision-making, productivity, interpersonal communication, organizational relationships^34^, job strain, perceptions of workplace social support^28^ and key leadership competencies including those related to decisiveness and creativity^35^. These are the reasons why nowadays mindfulness-based interventions are more and more recommended in the workplace.

The situation in which we observed the greatest increase in the use of online technologies for communication, training and every aspect of our daily life is the Covid-19 (COronaVIrus Disease 19, caused by the Severe Acute Respiratory Syndrome Coronavirus 2—SARS-CoV-2) pandemic. In Italy, some lockdowns limited to cities or regions had already started in February, but the nationwide lockdown started on the 9th of March, 2020, with an estimated 56 million people ordered to remain at home. Apart from the dreadful number of casualties and the enormous economic loss associated with the Covid-19 pandemic, the fear of contagion and the 2-months lockdown had a serious psychological impact on a large part of the Italian population, i.e., 40–50% of adults experiencing psychological distress^36^ and 30% of adults and children at risk for developing post-traumatic stress disorders^37^. Moreover, social distancing was previously proven to trigger negative mental health consequences, including intensified anxiety and depression^38,39^.

As a matter of fact, several studies assessing the efficacy of online mindfulness-based interventions during lockdown are emerging. An online mindfulness intervention significantly reduced perceived stress in Singaporean participants during lockdown^40^. The same study found comparable effects for online and in-person mindfulness training programs. Furthermore, online mindfulness training proved a reduction of anxiety and depression in Covid-19 patients themselves during isolation^41^, an increase of resilience in adolescents^42^, as well as employees’ sleep duration and work engagement^43^. Besides, several mindfulness-based protocols are currently being tested for their efficacy on Covid-19-related psychological symptoms^44–46^.

### Aim of the study

The aim of this study is to test the effectiveness of an online mindfulness-based training program on mindfulness, emotion regulation, mood, depression, anxiety, stress, resilience and sleep quality after the period of the first Italian lockdown. In fact, the mindfulness intervention was carried out during the period from the 19th of June to the 13th of August 2020, starting thus about 4 weeks after the end of the first Italian lockdown (which officially ended on the 18th of May 2020). During this period, many measures to prevent contagion were loosened as new cases significantly decreased in May. Nevertheless, the spectre of a second wave was starting to emerge during the end of summer, with a slow increase in new cases.

This time window was chosen because there is still scarce evidence about psychological consequences after the lockdown period, while the psychological effects during the lockdown were well investigated. One study on an Italian students sample found comparable psychopathological indices before and after the lockdown, with the worst depressive symptoms during the lockdown period and changes quickly vanishing after the lifting of lockdown^47^. Nevertheless, another study with a larger sample found that depression, stress, anxiety and fear of Covid-19 remained unchanged during and after the lockdown^48^. Another study from our group even found a worsening of several psychological well-being indices between the phases during and after the lockdown^49^. Therefore, after the lockdown, the psychological sequelae are still unclear and the effects of a mindfulness-based intervention during that period need to be investigated.

In particular, we investigated the effects of the mindfulness training program on a healthy population, specifically employees in a large-scale banking group. Given the increased distress employees have been experiencing during and after the Covid-19 lockdown(s) related to an overwhelming change in working paradigms (i.e., forced working from home) and in habits^50^, it is crucial to test the effectiveness of mindfulness interventions on this population since it is effortless and effective to administer it in an online modality on the (virtual) workplace^51^.

Based on the previous literature, specific hypotheses are related to (1) the increase in psychological well-being (i.e., mindfulness, use of mature emotion regulation strategies, positive affect, resilience) and decrease in perceived stress (i.e., use of basic emotion regulation strategies, negative affect, depression, anxiety, stress, insomnia) for participants undergoing the intervention, compared to the control group; (2) the protective value of mindfulness (i.e., no changes over time) when the control group experienced a worsening of psychological well-being and higher stress; (3) positive correlation between frequency of practice and indices of well-being, and difficulty perceived in practicing and indices of stress; negative correlation between frequency of practice and indices of stress, and difficulty perceived in practicing and indices of well-being.

## Results

### Power analysis

In order to identify the most adequate sample size, we performed an a-priori power analysis based on a meta-analysis on the effects of MBSR on healthy individuals^10^. We based the power analysis on the effect size the authors found for studies conducted by a facilitator with mindfulness training/experience (since our procedure respected this criterion): Hedge's *g* = 0.60. This effect size indicated the difference between the intervention and control groups in their changes before and after the intervention, i.e., the time × group interaction effect we investigated in our longitudinal analyses. The significance level was set to 0.05, test’s power was set to 0.85, with two-sample test and two-sided alternative hypothesis. This power analysis led to a result of n = 49. This criterion was respected in our final sample size: 69 participants in the intervention group and 63 in the control group (mean n = 66; actually corresponding to a power of 0.936 with the same parameters).

### Longitudinal analyses

Results from the longitudinal analyses are focused on the time × group interaction effects based on our a-priori hypothesis. Complete results including fixed main effects of time, group and covariates can be found in the Supplementary Information.

**Figure 1:**
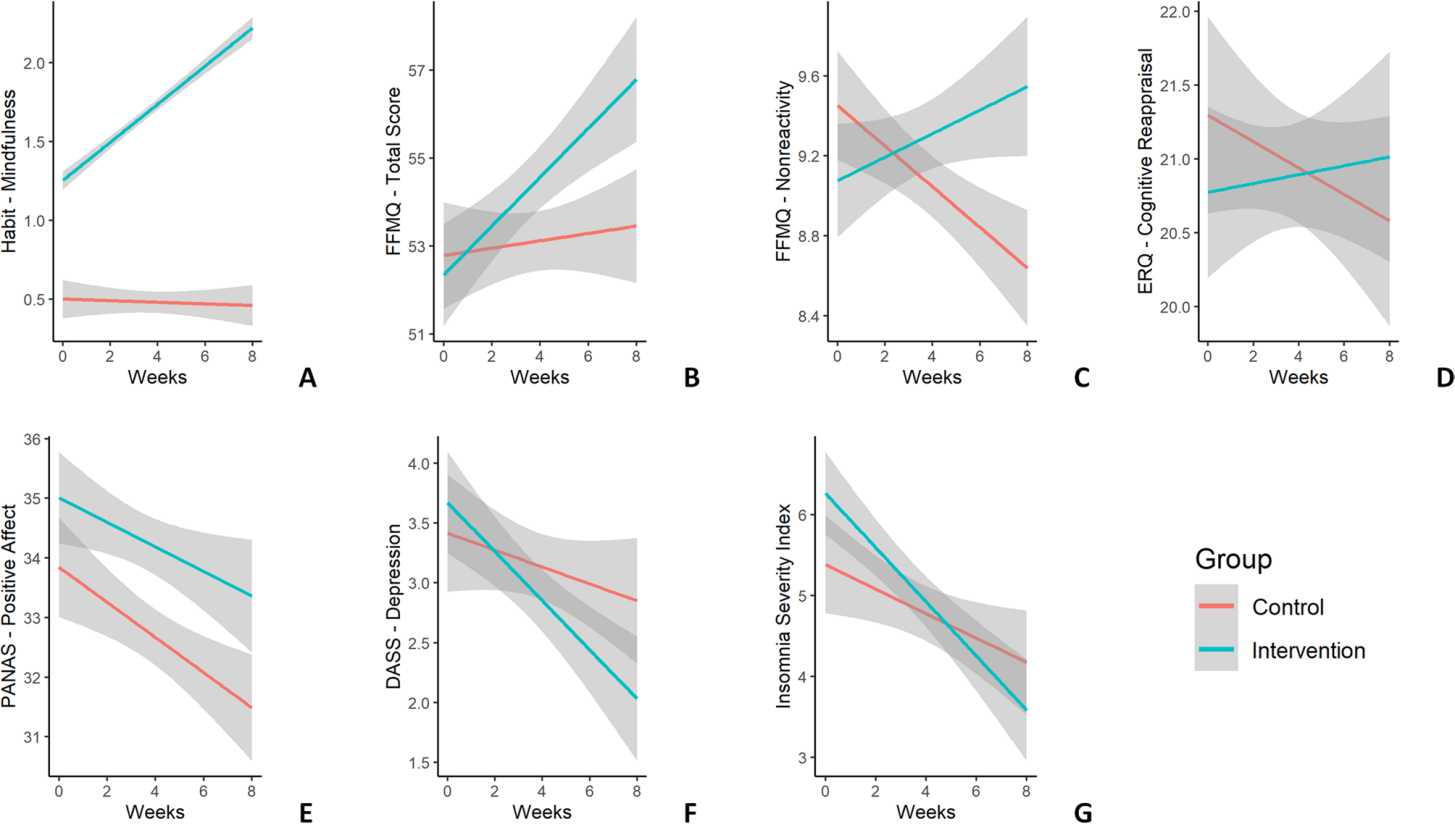
Time course of predicted average scores for different well-being and stress indices in longitudinal analyses. The x-axis in the plots represents the weeks of the intervention (from 0—before intervention—to 8—final week) and the y-axis represents participants’ scores. The red line represents the control group (N = 63), while the green line represents the intervention group (N = 69). The grey shaded area represents 95% confidence intervals. The y-axis represents predicted average scores for (**A**) the habit “Please specify how much time you spent doing the following activity: Practicing mindfulness (or other forms of meditation)”. See Supplementary Materials for further details on the questionnaire. (**B**) the mindfulness variable (FFMQ-15 questionnaire) total score and (**C**) the FFMQ-15 nonreactivity subscale, (**D**) the cognitive reappraisal variable (ERQ questionnaire), (**E**) the positive affect variable (PANAS questionnaire), (**F**) the depression variable (DASS-21 questionnaire), (**G**) the insomnia score (ISI questionnaire).

In the habits questionnaire, practicing mindfulness (or different forms of meditation) displayed a statistically significant time × group interaction effect: *F*(1, 112) = 44.30, *p* < 0.001 (Fig. 1A). The simple slope analysis proved that the intervention group showed a significant increase in the habit over time (*b* = 0.117, *t*(127) = 8.95, *p* < 0.001), while the control group did not display any significant change in time (*b* = − 0.0004, *t*(102) = − 0.03, *p* = 0.976). No other habits showed any significant time × group interaction effects (all *F*s < 3.3, all *p*s > 0.071), except for a significant effect in cooking (*F*(1, 112) = 4.35, *p* = 0.039). The simple slope analysis showed that control participants’ cooking habit decreased over time (*b* = − 0.068, *t*(103) = − 4.42, *p* < 0.001), while this did not happen in the intervention group (*b* = − 0.021, *t*(126) = − 1.28, *p* = 0.202).

The analysis on the FFMQ total score showed a statistically significant time × group interaction effect: *F*(1, 109) = 10.64, *p* = 0.001 (Fig. 1B). The simple slope analysis highlighted that the intervention group displayed a significant increase in FFMQ total score in time (*b* = 0.356, *t*(119) = 4.18, *p* < 0.001), whereas the control group showed no significant effect of time (*b* = − 0.024, *t*(103) = − 0.30, *p* = 0.764).

When testing specific FFMQ subscales, only the nonreactivity factor showed a statistically significant time × group interaction effect: *F*(1, 109) = 8.83, *p* = 0.004 (Fig. 1C). The simple slope analysis revealed that the control group exhibited a decrease in nonreactivity over time (*b* = − 0.117, *t*(102) = − 3.39, *p* = 0.001), while there was no statistically significant effect of time in the intervention group (*b* = 0.032, *t*(121) = 0.881, *p* = 0.380). Moreover, the nonjudging subscale showed a trend towards significance in the same interaction effect: *F*(1, 107) = 3.46, *p* = 0.065. However, the simple slope analysis showed a significant increase in nonjudgment over time in both groups (control: *b* = 0.102, *t*(100) = 3.41, *p* < 0.001; intervention: *b* = 0.184, *t*(119) = 5.72, *p* < 0.001). No other FFMQ subscales showed a significant time × group interaction effect (all *F*s < 2.9, all *p*s > 0.09).

In the ERQ questionnaire, the cognitive reappraisal subscale showed a trend towards statistical significance in the time × group interaction effect: *F*(1, 106) = 3.66, *p* = 0.058 (Fig. 1D). However, the simple slope analysis proved no statistically significant effect of time in either groups (control group: *b* = − 0.110, *t*(98.8) = − 1.90, *p* = 0.060; intervention group: *b* = 0.052, *t*(117) = 0.83, *p* = 0.408). The expressive suppression subscale did not present any significant time × group interaction effect: *F*(1, 93.9) = 1.32, *p* = 0.254.

When considering the PANAS scale results, the positive affect subscale exhibited a trend towards statistical significance in the time × group interaction effect: *F*(1, 106.08) = 3.72, *p* = 0.056 (Fig. 1E). The simple slope analysis showed a decrease in positive affect over time in the control group (*b* = − 0.367, *t*(98.7) = − 5.06, *p* < 0.001) and a smaller change in time in the same direction for the intervention group (*b* = − 0.163, *t*(118) = − 2.09, *p* = 0.039). The negative affect scale did not show a significant time × group interaction effect: *F*(1, 111) = 0.38, *p* = 0.845.

In the DASS questionnaire, the total score did not show a significant time × group interaction effect (*F*(1, 108) = 1.395, *p* = 0.240), but the depression subscale displayed a significant effect over the same interaction (*F*(1, 108) = 4.026, *p* = 0.047) (Fig. 1F). The simple slope analysis in this subscale revealed that depression scores decreased significantly over time in the intervention group (*b* = − 0.169, *t*(120) = − 3.59, *p* < 0.001), while they did not change significantly in the control group (*b* = − 0.041, *t*(100) = − 0.94, *p* = 0.350). Neither anxiety (*F*(1, 106) = 1.50, *p* = 0.224) nor stress subscales (*F*(1, 110) = 0.005, *p* = 0.943) showed any significant time × group interaction effects.

Neither RSA total score nor any of the subscales showed any significant time × group interaction effects over resilience: all *F*s < 1.6, all *p*s > 0.2.

In the ISI questionnaire, the time × group interaction effect was not statistically significant: *F*(1, 103) = 2.53, *p* = 0.115 (Fig. 1G). An exploratory simple slope analysis proved, however, a significant decrease in insomnia scores for the intervention group (*b* = − 0.245, *t*(114) = − 3.66, *p* < 0.001), while the control group did not show any significant changes in time (*b* = − 0.099, *t*(96.4) = − 1.56, *p* = 0.122).

### Correlations

All details for correlation results are reported in the Supplementary Information.

**Figure 2:**
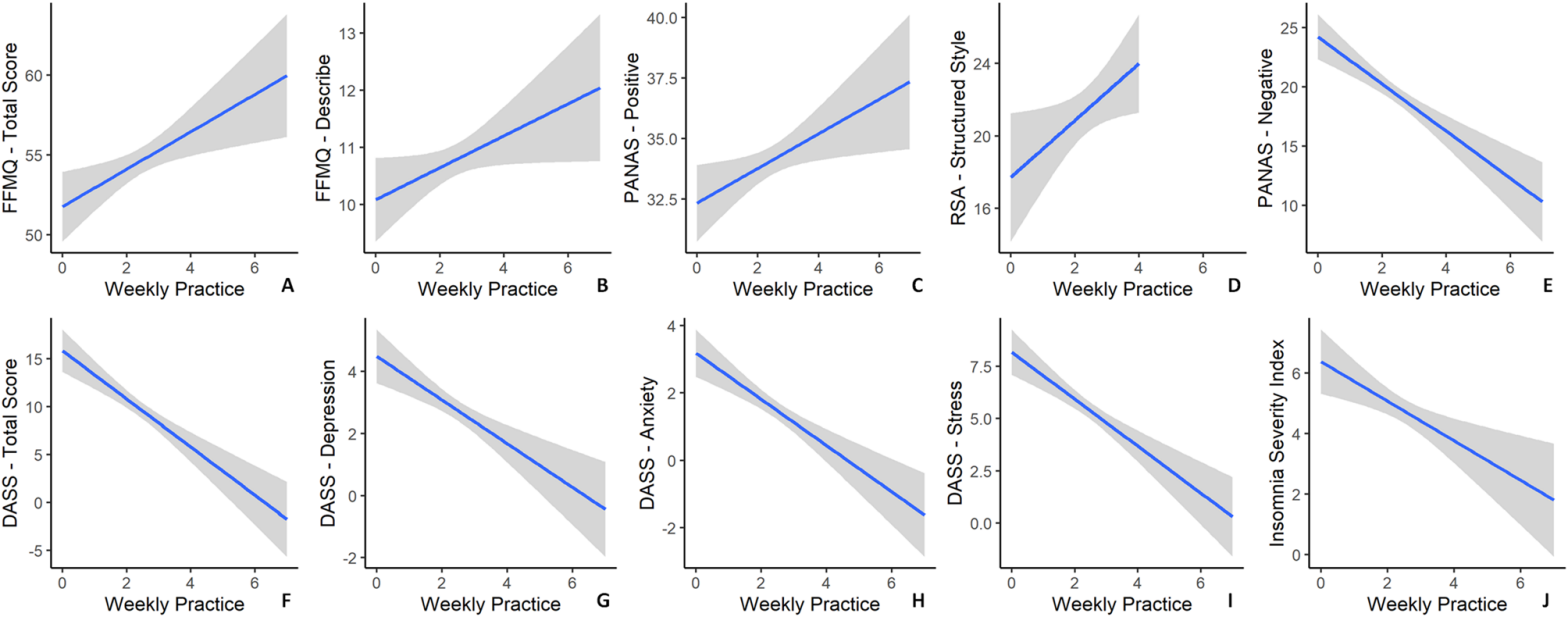
Plots showing significant correlations between the weekly frequency of mindfulness practice and several variables in the intervention group. The x-axis represents the weekly frequency of practice (ranging from 0 to 7 + times a week) and the y-axes represent (**A**) mindfulness total score (FFMQ-15), (**B**) describe subscale (FFMQ-15) (**C**) positive affect (PANAS), (**D**) structured style subscale (RSA) (participants only presented 0 to 4 values on the x-axis in this subsample), (**E**) negative affect (PANAS), (**F**) DASS-21 total score, (**G**) depression subscale (DASS-21), (**H**) anxiety (DASS-21), (**I**) stress subscale (DASS-21), (**J**) insomnia (ISI). The grey shaded area represents 95% confidence intervals.

In the intervention group, the frequency of practice showed statistically significant positive correlations with several well-being indices (FFMQ total score, FFMQ—Describe, PANAS—positive affect, RSA—structured style) and negative correlations with distress indices (PANAS—negative affect, DASS total score, DASS—depression, DASS—anxiety, DASS—stress, ISI) (Fig. 2).

**Figure 3:**
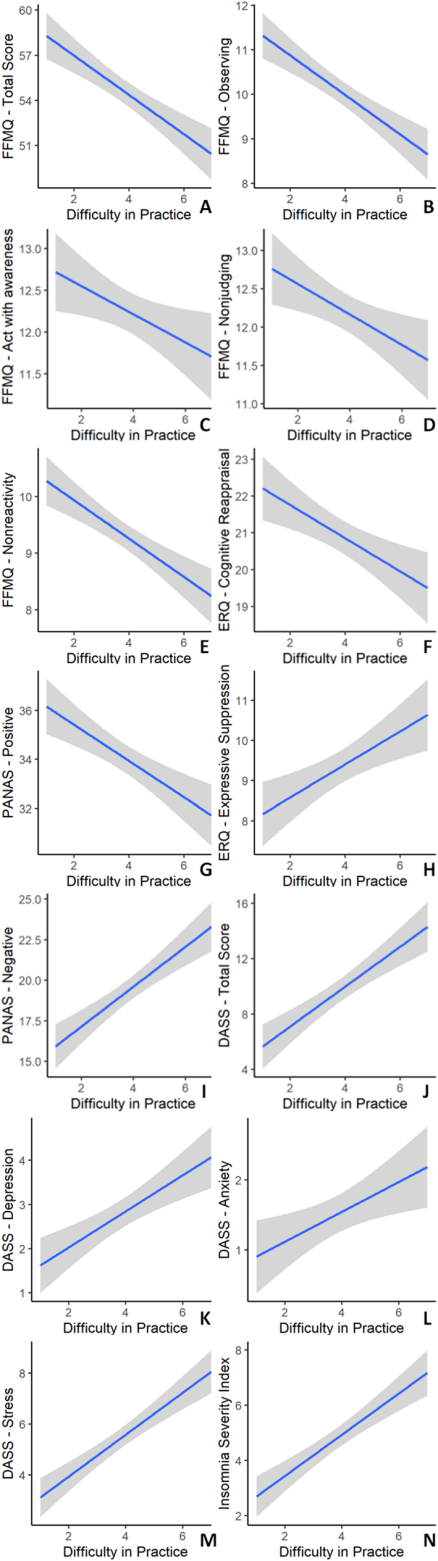
Plots showing significant correlations between the difficulty perceived in weekly mindfulness practice and several variables in the intervention group. The x-axis represents difficulty perceived in weekly practice (ranging from 1—very easy to 7—very difficult) and the y-axes represent (**A**) mindfulness total score (FFMQ-15), (**B**) observing subscale (FFMQ-15), (**C**) acting with awareness subscale (FFMQ-15), (**D**) nonjudging subscale (FFMQ-15), (**E**) nonreactivity subscale (FFMQ-15), (**F**) cognitive reappraisal subscale (ERQ), (**G**) positive affect (PANAS), (**H**) expressive suppression subscale (ERQ), (**I**) negative affect (PANAS), (**J**) DASS-21 total score, (**K**) depression subscale (DASS-21), (**L**) anxiety subscale (DASS-21), (**M**) stress subscale (DASS-21), (**N**) insomnia (ISI). The grey shaded area represents 95% confidence intervals.

Conversely, the difficulty perceived in weekly practice was negatively correlated with well-being indices (FFMQ total score, FFMQ—observing, FFMQ—acting with awareness, FFMQ—nonjudging, FFMQ—nonreactivity, ERQ—cognitive reappraisal, PANAS—positive affect) and positively correlated with distress indices (ERQ—expressive suppression, PANAS—negative affect, DASS total score, DASS—depression, DASS—anxiety, DASS—stress, ISI) (Fig. 3).

As a final check, we found a statistically significant strong negative correlation between the frequency of practice and the difficulty perceived in weekly practice: r = − 0.521, p < 0.001.

## Discussion

Mindfulness training programs were proven to be effective in improving well-being and reducing perceived stress in several populations (especially those prone to burnout) and conditions^8–10^. These effects were also found in online training, in particular during Covid-19 lockdowns by some preliminary results^40,52^. The aim of this study was to test the effectiveness of an online mindfulness training program after the first Italian Covid-19 lockdown, when the measures to prevent contagion were loosened, but the second wave was starting to emerge. Indeed, the psychological sequelae after the lockdown period are still to be clarified^47,48^. We found significant differences between the intervention group and the control group over time in the measures of mindfulness (in particular the nonreactivity subscale), positive affect, depression and insomnia. Moreover, we found that the frequency of practice and the ease perceived in practicing were positively correlated to several indices of well-being (mindfulness, positive affect, cognitive reappraisal) and negatively correlated to several indices of stress (negative affect, depression, anxiety, stress, insomnia, expressive suppression).

The mindfulness-based intervention showed a positive effect in time on several indices, compared to no changes in time for the control group (practicing mindfulness habit, mindfulness, depression, and marginally insomnia). These results confirm our first hypothesis (i.e., the increase in psychological well-being and decrease in perceived stress for participants undergoing the intervention, compared to the control group). First of all, the difference in practicing mindfulness acted as a double-check and showed the compliance of participants in the intervention group (i.e., participants in the intervention group showed an increase in how often they practiced during the program, while participants in the control group did not; see also section “Participants”). The effects of mindfulness training on mindfulness, depression and insomnia are now quite renowned^8,10,21,53^ and the mechanism guiding all these changes seems to be the increase in mindful attitude towards one’s own experience, in particular in terms of acceptance, nonreactivity and nonjudgment^54–57^. It is crucial to highlight that these changes were demonstrated also in the period after a lockdown, a time ruled by uncertainties during which people were conflicted between hope and resignation. These results prove that abilities promoted by mindfulness-based interventions can overcome the peculiar psychological conditions reflecting the unprecedented health and social situation. Moreover, these improvements cannot be attributed to the lockdown lifting, given their absence in the control group.

Two indices of well-being (the nonreactivity mindfulness subscale and marginally positive affect) significantly decreased over time in the control group, while they showed no significant differences over time in the intervention group. These results confirm our second hypothesis (i.e., the protective value of mindfulness when the control group experienced a worsening of psychological well-being and higher stress). They are particularly relevant, especially considering the abovementioned importance of nonreactivity in driving the effects of the intervention on well-being^56^ and because they reflect how the specific societal context was influencing people in that specific post-lockdown time. The latest intervention weeks corresponded to a small but constant increase in new cases in Italy, prospecting thus the arrival of a second wave in the pandemic. Together with the distress accumulated during the previous months, this could explain the increase in emotional reactivity since hope was giving way to fear and stress related to the perspective of new restrictions and collective danger to people’s health. The first lockdown represented a traumatic experience for many people who experienced it^37^, and the idea of a second wave meant uncertainty and led to the fear of the impossibility of emerging from the pandemic. This particular and decisive phase was not investigated in detail in the previous literature. The absence of a temporal change in the intervention group showed the protective value of the mindfulness intervention: the program promoted well-being, protected participants from the effects of external stressors and helped them cope with uncertainty and fear. Moreover, we can be confident enough in stating that this difference was due to the intervention, given the absence of differences between groups in the use of habits as coping strategies.

Correlation tests in the intervention group showed that the frequency of practice was positively correlated with well-being indices (mindfulness—total score and describe subscale—positive affect, structured style in resilience) and negatively correlated with several stress indices (negative affect, DASS—total score and all subscales—insomnia), confirming thus our third hypothesis. The positive effect of mindfulness practice on these variables is well known^10^, with particular respect to the frequency of practice. Previous literature robustly showed that the time spent in home practice is significantly correlated with the extent of improvement in mindfulness and several indices of well-being also in standard in-person MBSR protocols^58,59^. The result on resilience is too narrow (i.e., significant correlation only on one subscale, no further significant results in group differences) to be interpreted, even though mindfulness-based interventions showed a positive effect on resilience in previous literature^42^. Furthermore, perceived difficulty in practicing was negatively correlated with well-being indices (mindfulness—total score and observing, acting with awareness, nonjudging, nonreactivity subscales—cognitive reappraisal strategy, positive affect) and positively correlated with several stress indices (expressive suppression strategy, negative affect, DASS—total score and all subscales—insomnia). In this case, it is not easy to test a causal effect, also given the bidirectional nature of correlation tests. Indeed, it is arduous to discern whether higher stress made the practice more difficult for participants, or experiencing more difficulties in practicing caused higher stress. Nevertheless, in both cases, these results show the intrinsic relationship between perceived stress and perceived mindfulness in everyday life. A focus on emotion regulation strategies is crucial: we reported that higher difficulty perceived in practice is related to more frequent use of basic strategies to regulate emotions (i.e., expressive suppression) and less frequent use of advanced strategies (i.e., cognitive reappraisal). This result is in line with previous literature showing analogous results^17,18^ and supports thus the hypothesis of overlap between “mindfulness” and “emotion regulation” theoretical constructs^14^, even though cognitive reappraisal strategies are never explicitly taught in mindfulness practices^16^.

Since we focused our online program on a specific healthy population, i.e., employees in a large-scale banking group, it is crucial to take into account the impact of this study on workplace applications. Mindfulness-based interventions have shown a relevant efficacy on workers’ well-being^3^ and organizational parameters^34^, in both in-person and online modalities^33^. Given the increased distress employees have been experiencing during the Covid-19 pandemic^50^, caused by a critical change in working paradigms, our study showed that administering online programs aimed at coping with stress on this population is crucial now more than ever. Indeed, this type of intervention is recommended in the workplace, given its critical effects on well-being in this period dominated by uncertainty. Therefore, it is crucial that employers promote and encourage initiatives of this kind in small, medium, and large-scale companies. In this respect, companies should acknowledge that this category of training can now be performed mostly in an online modality and the internet is being overused to work from home, often leading to an increase in techno-stress.

From this perspective, the results that emerged from this study are of particular relevance and novelty when considering the historical period during which they were collected. In the pandemic situation, the internet is abused in every aspect of life (e.g., education, work, personal relationships). At this time, we were living a sort of technological/social paradox: in-person social contact is impossible due to restrictions and the risk of Covid-19 transmission and, yet, we do not wish to use the internet to keep in touch with friends and relatives, given the techno-stress related to the ubiquity of this medium in our life^60,61^. For this reason, online mindfulness-based interventions might have led to an increase in distress related to technology overuse instead of a decrease. On the contrary, our results showed that the protective value of mindfulness was stronger than technology overuse-related stress.

Concerning mindfulness-based interventions, considering and discussing some methodological aspects is of paramount importance, especially given the reproducibility and replicability crisis in psychological science we are experiencing^62^. In the first place, the duration typically suggested in mindfulness programs is at least 8 weeks^1^. De facto, an interesting study proved that an 8-week mindfulness-based intervention can induce neurofunctional changes similar to those observed in traditional long-term meditation practice^63^.

A further important aspect is related to home practice: besides the sessions with a trainer, the home practice was proven to be a significant mediator influencing the outcome of training in MBSR^59^. Indeed, a study focused on the MBSR program showed that the time spent engaging in home practice of formal meditation exercises was significantly related to the extent of improvement in most facets of mindfulness and several measures of symptoms and well-being^58^. For this reason, we used minimum practice frequency as an exclusion criterion in the intervention group. The presence of trainers for weekly practice presentations is another crucial aspect of online interventions that was acknowledged in our research since guided online mindfulness-based interventions showed a larger effect size compared to unguided ones^24^.

Finally, though several indices of well-being increase thanks to mindfulness practices, a caveat must be made. Mindfulness meditation must not be regarded as a therapeutic panacea for all ailments, and the effects of mindfulness practice on health appear similar in magnitude to the changes demonstrated by other conventional approaches for treating stress, pain, and illness, including the administration of psychoactive medications, psychotherapy, health education, and behaviour modification^2,64^. Unfortunately, with the increase of interest in mindfulness, some studies appeared to present spurious results with poor methodology^65^. Therefore, it is crucial to use clear and rigid experimental methods to draw solid conclusions, starting with adequate an sample size, longitudinal design and collecting data from an adequate control group.

### Limitations

The main limitation of this study is the lack of a follow-up measure to test whether the effects of the online program lasted in time through the second Covid-19 wave. This could be a crucial aspect to investigate the long-term effects of mindfulness-based interventions and practice maintenance after the program ended. This element will be investigated in future research. A further limitation to the generalizability of this study is the lack of a direct comparison with an in-person mindfulness program. However, recent literature^40^ compared online vs. in-person mindfulness-based interventions during Covid-19 lockdown, finding comparable effects on stress from the two categories of training. A final limitation is represented by the fact that the group sampling was not fully randomised. Indeed, as specified in section “Participants”, 28 participants recruited from outside of the banking group were included in the control group to balance sample sizes since the initial response from the (no-treatment) control group was extremely lower than from the intervention group. Although we co-varied participants’ origin (i.e., banking group vs. students and acquaintances) in the longitudinal analyses to exclude the effect of this factor, this difference could possibly bias our results, which need to be replicated in future studies.

### Conclusions

In this study, we investigated the effects of an online mindfulness-based intervention after the Italian Covid-19 lockdown with adult participants working in the banking industry. We found a positive and protective value of the mindfulness practice over time on mindfulness, positive affect, depression, and insomnia. Moreover, the frequency of practice and the ease perceived in practicing were positively correlated to several indices of well-being and negatively correlated to several stress indices. These results demonstrated the extremely positive effects of mindfulness practice on well-being and stress, especially in a psychologically challenging period such as the Covid-19 lockdown and post-lockdown. For this reason, mindfulness programs should be spread and promoted online during this period, especially in the workplace, as it could be helpful in several aspects of psychophysical well-being.

## Methods

### Participants

One hundred and thirty-two participants took part in the experiment on a voluntary basis. The only inclusion criterion was the absence of any psychiatric record. These participants were recruited among employees in a large-scale banking group, post-graduate university students, and acquaintances.

The sample size was identified in n = 49 by using an a-priori power analysis based on a previous meta-analysis (see section “Power analysis”). Initially, participants from the banking group were randomly assigned to two groups of seventy participants each. Nevertheless, the response from these two groups was very different; sixty-nine participants from the intervention group completed at least one weekly survey but only thirty-five participants from the (no-treatment) control group did so. Therefore, participants recruited from outside of the banking group (i.e., students and acquaintances, n = 28) were included in the control group to balance sample sizes. This led us to obtain two balanced groups: intervention group (n = 69; 44 F, mean ± sd age 44.7 ± 8.9) and (no-treatment) control group (n = 63; 42 F, mean ± sd age 38.3 ± 10.4).

Volunteers were paid 19.50€ for their participation if they completed the 8-week surveys. In order to control for possible biases related to participants’ origin, in all longitudinal analyses participants’ origin was co-varied. In addition, these volunteers were blind to the real aim of the study until the final debriefing (in order to avoid possible biased responses).

An Intention-To-Treat analysis approach^66^ was used, i.e., data from all participants were included independently of the number of surveys completed. This approach allows having a conservative estimation of the treatment effect and avoiding biases related to drop-outs and participants’ adherence.

To verify the intervention's effectiveness, we added two further criteria for potential exclusion. In the intervention group, we checked that each participant’s average weekly frequency of practice was at least 1. No participants were excluded according to this criterion (min value = 1.00). In the control group, we checked that participants’ habit of practicing mindfulness or other forms of meditation was lower than in the intervention group (by using the habits questionnaire, see section “Materials” and Supplementary Information). Five participants in the control group showed an average value greater than or equal to 2 (i.e., “few times a week”). Focusing on these five participants, we checked that their habit of practicing meditation was not increasing between the starting survey and the following 8 weeks. By using a mixed linear regression with data from these participants, we found no significant effect of time on their frequency of practice (*b* = − 0.05, *t* = − 1.381, *p* = 0.176). Therefore, we established that these five participants did not change their habits during the 8 weeks of data collection and were not excluded. Moreover, we found a clear increase in the frequency of practice in the intervention group due to the training program exploit, but no significant increase in the control group (see section “Results”). Accordingly, we can conclude that the habits of these five participants reflect the results found on average in the control group.

#### Ethical statement

All participants were provided with an exhaustive description of all the experimental procedures and were required to sign a written informed consent before taking part in the study. The study was conducted in accordance with the ethical standards laid down in the 1964 Declaration of Helsinki and under a protocol approved by the Area Vasta Nord Ovest Ethics Committee (protocol n. 24579/2018).

### Procedure

The starting survey was administered during the week 11th–18th of June 2020, about three weeks after the end of the first Italian lockdown (officially ended on the 18th of May 2020). All surveys were filled in online on the Google Form platform and were accessed by using links sent by the experimenter. In the first part of the starting survey, we asked for sex, age and habits. Habits were investigated by using an ad-hoc questionnaire aimed at studying the use of habits as coping strategies towards stress. In the second part, several validated questionnaires were administered to investigate different constructs related to well-being and stress: 15-item Five Facet Mindfulness Questionnaire (FFMQ-15^67^); Emotion Regulation Questionnaire (ERQ^68^); Positive And Negative Affect Scale (PANAS^69^); Depression Anxiety Stress Scale-21 (DASS-21^70^); Resilience Scale for Adults (RSA^71^); Insomnia Severity Index (ISI^72^). See the following section for more details on each questionnaire. The average completion time was around 15 min.

During the following eight weeks (19th of June–13th of August 2020), participants in the intervention group underwent the mindfulness training program and all participants filled in the intermediate survey on a weekly basis. The intermediate survey included a subsample of the questionnaires in the starting survey, related to state variables: habits, FFMQ-15, ERQ, PANAS, DASS-21, and ISI. Only in the intervention group, participants were also asked to indicate their weekly frequency of mindfulness practice (from 0 to 7 + sessions) and how difficult they perceived their practice (on a Likert scale ranging from 1—very easy to 7—very difficult). The average completion time was around 10 min.

The mindfulness training program lasted for 8 weeks and was administered online only to the intervention group. Eight different practices were chosen based on the MBSR protocol^1^, by alternating sitting and moving practices (see Supplementary Information for details). At the start of each week, the weekly practice was presented by two mindfulness trainers on a conference call with the intervention group. The two trainers were trained and instructed in administering the MBSR protocol and in further contemplative practices (i.e., vipassana meditation, yoga, tai-chi chuan). Participants in this group could take part in the weekly call on a voluntary basis. Their privacy was protected by participating anonymously and with no possibility to turn their webcams on. After the practice, participants could ask the trainers any questions about mindfulness practice and their own experience during the online program. The experimenters sent an audio or video guide for the weekly practice (accessible via a link, average duration: 20 min) every week. Participants were asked to practice using the guide in a protected environment at least three times per week.

After the eighth week of intervention, all participants had to fill in the final survey. This survey contained the same questionnaires as the intermediate survey and the RSA. The RSA measures a stable variable (i.e., resilience), therefore we decided to administer it only in the starting and final surveys to assess the difference before and after the program. Thirty-six participants in the intervention group and forty-nine in the control group filled in the final survey. Hence, we could have a measure of the difference in the RSA only for this sub-group of participants. The average completion time was around 15 min.

Participants in the control group were asked to fill in all the weekly surveys (from week 0 to 8) without taking part in the training.

### Materials

#### Habits

The habits questionnaire investigated the weekly frequency of 22 different habits used as coping strategies towards stress (e.g., web browsing, cooking, watching movies/series, physical activity). The response to each habit could be: 0: “I do not carry out this activity”, 1: “once a week or less”, 2: “few times a week”, 3: “less than one hour a day”, 4: “one to three hours a day”, 5: “more than three hours a day”. See Supplementary Information for the full questionnaire.

#### Mindfulness

Mindfulness was investigated by using the 15-item Five Facet Mindfulness Questionnaire (FFMQ-15^67^). Each item is scored on a 5-point Likert scale (from 1 = “Never or very rarely true”, to 5 = “Very often or always true”). Items were scored into five subscales: observing (attending to sensory stimuli that mainly derive from external sources and the body as well as related cognitions and emotions), describing (labelling internal experiences with words), acting with awareness (ongoing attention to, and awareness of present activity and experience), nonjudging (having a non-evaluative attitude towards one’s thought and emotional processes while focusing on inner experiences, rather than taking on a critical stance), and nonreactivity (assuming a stance that implies being able to perceive thoughts and feelings, especially when they are distressing, but without feeling compelled to react or being overwhelmed). Single items were translated into Italian by using the Italian complete version of the questionnaire^73^. The Italian version of the FFMQ showed good to excellent internal consistency as a whole (alpha = 0.86) with sub-scale consistency ranging from 0.65 to 0.81, test–retest stability for the total score being 0.71, and a good concurrent validity as demonstrated by significant correlations between the FFMQ scores and several self-report measures related to mindfulness^73^.

#### Emotion regulation

Participants’ use of different emotion regulation strategies was investigated with the Emotion Regulation Questionnaire (ERQ^68^). This is a 10-item questionnaire, in which each item is scored on a 7-point Likert scale (from 1 = “Strongly disagree” to 7 = “Strongly agree”). Items are scored into two separate subscales investigating expressive suppression (basic emotion regulation strategy, i.e., suppressing the behavioural expression of the emotion) and cognitive reappraisal (more advanced cognitive emotion regulation strategy, aimed at modifying the internal representation of an event to change one’s own emotional experience)^19^. Previous literature (*ibidem*) showed that people who use cognitive reappraisal more often tend to experience and express greater positive emotion and lesser negative emotion, whereas people who use expressive suppression experience and express lesser positive emotion, yet experience greater negative emotion. Both subscales showed high internal consistency reliability (Alpha values ranging from 0.68 to 0.80 across four different samples) and test–retest reliability across 3 months was 0.69 for both scales^19^.

#### Positive and negative affect

Participants’ affect was recorded by using the Positive And Negative Affect Scale (PANAS^69,74^). Given the weekly administration, we used the PANAS with the “week” time instruction, i.e., each participant was asked to rate to what extent s/he felt as specified by each item during the last week. Each of the 20 items is scored on a 5-point Likert scale (from 1 = “very slightly or not at all” to 5 = “extremely”). Half of the items constituted the positive affect subscale, whereas the remaining half constituted the negative affect subscale. The alpha internal consistency reliability indices were shown to be acceptably high, ranging from 0.86 to 0.90 for positive affect and from 0.84 to 0.87 for negative affect. Test–retest reliability showed no significant differences across an 8-week interval^74^.

#### Depression, anxiety and stress

Perceived depression, anxiety and stress were measured using the Depression Anxiety Stress Scale-21 (DASS-21^70^). It is a 21-item self-report questionnaire assessing core symptoms of anxiety, depression and stress. Each item is scored on a 4-point Likert scale (ranging from 0 = “Did not apply to me at all over the last week” to 3 = “Applied to me very much or most of the time over the past week”). The DASS-21 has been shown to have good psychometric properties, i.e., internal consistency: Cronbach's alphas were 0.94 for Depression, 0.87 for Anxiety, and 0.91 for Stress; concurrent validity indices above 0.60 with several other inventories) both in clinical and non-clinical samples^75^, and contains three subscales: Depression, Anxiety, and Stress.

#### Resilience

The Resilience Scale for Adults (RSA^71,76^) measures six resilience protective factors, of which four are intrapersonal factors (personal strength, planned future, social competence, and structured style) and two are interpersonal factors (family cohesion and social resources). This scale comprises 33 items, scored along a 7-point semantic differential scale. This questionnaire was administered only in the starting and final surveys, given its construct stability in time (*ibidem*). From a psychometric perspective, the internal consistency of the subscales of the RSA was satisfactory, ranging from 0.67 to 0.90. The test–retest correlations were all satisfactory for the subscales of RSA, ranging from 0.69 to 0.84 (*p* < 0.01)^76^.

#### Insomnia

The Insomnia Severity Index (ISI^72,77^) is a 7-item self-report brief questionnaire designed to assess the severity of insomnia symptoms and sleep disorders. Given the weekly frequency, questions were referred to the last week and participants were asked to report the severity of symptoms on a 5-point Likert scale (ranging from 0 = “no problem” to 4 = “very severe problem”). Sleep was proven to be affected by anxiety/stress levels and to be modulated by mindfulness practice^21,22^. ISI internal consistency was excellent for both clinical and non-clinical samples (alpha of 0.90 and 0.91). Convergent validity was supported by significant correlations between total ISI score and measures of fatigue, quality of life, anxiety, and depression^72^.

### Statistical analyses

In longitudinal analyses, the time course of each variable of interest (i.e., specific habits and the subscales from each questionnaire) was analysed by using mixed-effects linear models. In each model, the variable of interest was used as the dependent variable, while time and group (2-level factor: intervention vs. control) were used in interaction as fixed effects. Time was coded as a continuous variable, representing the weeks of training (therefore ranging from 0 to 8). Participants’ origin (2-level factor: banking group vs. students and acquaintances) was co-varied in all longitudinal analyses to control for possible biases related to this variable. Since a significant difference between the intervention group and the control group was found in the FFMQ—Acting with awareness variable in t0 (see Supplementary Information), we also co-varied this variable in all longitudinal analyses to investigate whether group differences had an influence on the results. Random intercept and time effect were computed on each participant. As a matter of fact, the multilevel nature of mixed-effects models allowed us to fit a regression line for each participant in each variable, and then compare the time course (i.e., the slope of the regression line) between groups^78^. Degrees of freedom in mixed-effects models were computed using Satterthwaite's approximation.

The main effect of interest was the two-way interaction time × group, as this effect represents a different trend over time for the intervention group compared to the control group. When this interaction effect was found as statistically significant, a simple slope analysis was performed in order to test whether the time slope was significantly different from zero in the two groups. The simple slope analysis was performed also when the inferential tests suggested a trend towards a difference between the two groups (*p* < 0.06) since we a priori hypothesized differences over time between the two groups and these trends could suggest their presence. Moreover, when testing planned comparisons, the interaction effects probed by using simple slope analyses do not necessarily need to be statistically significant if comparisons are based on a priori hypotheses^79^. To control for Type I error, all inferential tests in simple slope comparisons were corrected by using Tukey’s HSD method.

Moreover, we made a specific focus on participants in the intervention group to investigate what indices of well-being are most affected by the frequency of practice and difficulty perceived in practice. When considering data from the intervention group in intermediate and final surveys (i.e., weeks from 1 to 8), we computed a Pearson’s correlation matrix between the frequency of practice (and difficulty perceived in practicing) and all the state variables we recorded in the questionnaires (i.e., FFMQ-15, ERQ, PANAS, DASS-21, RSA, ISI). All correlation tests are corrected for multiple comparisons by using Tukey’s HSD method.

All statistical analyses were performed in RStudio software^80^. Power analysis was performed by using the *pwr* package^81^; mixed-effects models were estimated by using the *lme4* and *lmerTest* packages^78,82^; simple slope analyses were performed with the *gamlj* package^83^, based on *emmeans*^84^; plots were created using the *ggplot2* package^85^.

## Supplementary Information

Supplementary Information.

## Data Availability

Data and materials will be made available by the authors upon request, without undue reservation.
