# Supplementary material for: Mindfulness-based online intervention increases well-being and decreases stress after Covid-19 lockdown
Source: Sci Rep. 2022 Apr 20;12:6483. doi: 10.1038/s41598-022-10361-2 (PMC9019542; doi:10.1038/s41598-022-10361-2)
Supplement: Supplementary file 1 — Supplementary Information. [file 41598_2022_10361_MOESM1_ESM.docx]

# Supplementary Materials

## Habits questionnaire

**Let’s talk about your daily routine during LAST WEEK. Please specify how much time you spent doing the following activities:**

|  | **0**  **I do not carry out this activity** | **1**  **Once a week or less** | **2**  **Few times a week** | **3**  **Less than one hour a day** | **4**  **One to three hours a day** | **5**  **More than three hours a day** |
| --- | --- | --- | --- | --- | --- | --- |
| Web browsing |  |  |  |  |  |  |
| Playing cards or board games |  |  |  |  |  |  |
| Watching movies or TV series |  |  |  |  |  |  |
| Watching YouTube videos |  |  |  |  |  |  |
| Doing high-intensity workout (running, weights, rope) |  |  |  |  |  |  |
| Practicing yoga/pilates |  |  |  |  |  |  |
| Practicing mindfulness (or other forms of meditation) |  |  |  |  |  |  |
| Watching television |  |  |  |  |  |  |
| Reading news |  |  |  |  |  |  |
| Listening to podcast |  |  |  |  |  |  |
| Reading a book |  |  |  |  |  |  |
| Listening to the radio |  |  |  |  |  |  |
| Social networking (Facebook, Instagram, etc) |  |  |  |  |  |  |
| Calling friends/family |  |  |  |  |  |  |
| Painting / drawing / colouring |  |  |  |  |  |  |
| Listening to music |  |  |  |  |  |  |
| Playing a musical instrument |  |  |  |  |  |  |
| Learning through online courses |  |  |  |  |  |  |
| Cooking |  |  |  |  |  |  |
| Listening to audiobooks |  |  |  |  |  |  |
| Playing videogames |  |  |  |  |  |  |
| Walking the dog |  |  |  |  |  |  |

## Mindfulness training

- **Week 1:** *Awareness of breath (short)*10-min sitting practice. Participants’ main focus of attention is the physical sensations of breathing.
- **Week 2:** *Mindful yoga*20-min moving practice. Participants perform specific movements (while standing, sitting or lying on a yoga mat) from the yogic tradition and focus their attention on the physical sensations related to the movements.
- **Week 3:** *Body scan*25-min lying (or sitting) practice. Participants slowly explore the physical sensations from all body districts.
- **Week 4:** *Walking meditation*
  20-min moving practice. Participants focus their attention on the physical sensations in their bodies while performing a slow walk.
- **Week 5:** *Ten mindful movements*
  15-min moving practice. Participants perform specific movements (while standing) taught by the Zen Buddhist monk Thich Nhat Hanh and focus their attention on the physical sensations related to the movements. Ref: <https://youtu.be/4mz-dJFkmrk>
- **Week 6:** *Awareness of breath (long)*10/20/30-min sitting practice (i.e., the guided meditation signals the end of 10 and 20 minutes and participants are free to choose when to stop). Participants’ main focus of attention is the physical sensations of breathing.
- **Week 7:** *Awareness of breath, sounds and thoughts*
  10/20/30-min sitting practice (i.e., the guided meditation signals the end of 10 and 20 minutes and participants are free to choose when to stop). Participants’ main focuses of attention are, sequentially, the physical sensations of breathing, perceived sounds and the flow of one’s own thoughts.
- **Week 8:** *Loving-kindness meditation*
  20-min sitting practice. Participants’ aim is to direct loving-kindness sentences and intentions towards themselves and towards significant ones.

# Adherence table

**Table representing participants’ adherence to the weekly surveys.**

**The column “General Adherence” shows how many participants completed each weekly survey out of the initial sample who volunteered for the study.**

| WEEK | GROUP | GENERAL ADHERENCE |
| --- | --- | --- |
| 0 | Intervention | 68/69 |
|  | Control (employees) | 34/35 |
|  | Control (acquaintances) | 28/28 |
| 1 | Intervention | 62/69 |
|  | Control (employees) | 31/35 |
|  | Control (acquaintances) | 27/28 |
| 2 | Intervention | 57/69 |
|  | Control (employees) | 34/35 |
|  | Control (acquaintances) | 26/28 |
| 3 | Intervention | 53/69 |
|  | Control (employees) | 30/35 |
|  | Control (acquaintances) | 25/28 |
| 4 | Intervention | 45/69 |
|  | Control (employees) | 31/35 |
|  | Control (acquaintances) | 26/28 |
| 5 | Intervention | 43/69 |
|  | Control (employees) | 29/35 |
|  | Control (acquaintances) | 25/28 |
| 6 | Intervention | 44/69 |
|  | Control (employees) | 28/35 |
|  | Control (acquaintances) | 24/28 |
| 7 | Intervention | 32/69 |
|  | Control (employees) | 24/35 |
|  | Control (acquaintances) | 25/28 |
| 8 | Intervention | 36/69 |
|  | Control (employees) | 25/35 |
|  | Control (acquaintances) | 24/28 |

# Full results from Longitudinal analyses

**Results from full Analysis of Variance (ANOVA) are reported for each model discussed, for complete transparency.**

All analyses included the following independent variables:

- Time (numerical, representing training weeks from 0 to 8)
- Group = experimental groups (factor, intervention vs. control)
- Origin group (factor, banking group vs. students and acquaintances)
- FFMQ – Act with awareness (numerical, covariate)

**Statistically significant effects are in bold.**

## Habit – Mindfulness practice

|  | **NumDF** | **DenDF** | **F value** | **P value** |
| --- | --- | --- | --- | --- |
| **Time** | **1** | **117.73** | **42.4749** | **< .001** |
| **Group** | **1** | **141.56** | **21.4472** | **< .001** |
| Origin group | 1 | 122.00 | 0.0040 | 0.9499 |
| FFMQ – Act with awareness | 1 | 716.72 | 0.2314 | 0.6306 |
| **Time * Group** | **1** | **111.87** | **44.3008** | **< .001** |

## Habits – Cooking

|  | **NumDF** | **DenDF** | **F value** | **P value** |
| --- | --- | --- | --- | --- |
| **Time** | **1** | **117.31** | **15.3595** | **< .001** |
| Group | 1 | 139.84 | 0.3402 | 0.56066 |
| Origin group | 1 | 123.49 | 1.0511 | 0.30727 |
| FFMQ – Act with awareness | 1 | 862.76 | 1.2653 | 0.26096 |
| **Time * Group** | **1** | **111.60** | **4.3485** | **0.03932** |

## FFMQ – Total score

|  | **NumDF** | **DenDF** | **F value** | **P value** |
| --- | --- | --- | --- | --- |
| **Time** | **1** | **112.68** | **7.9584** | **0.005658** |
| **Group** | **1** | **129.55** | **5.0712** | **0.026009** |
| **Origin group** | **1** | **124.09** | **5.3168** | **0.022780** |
| **FFMQ – Act with awareness** | **1** | **918.49** | **294.6676** | **< .001** |
| **Time * Group** | **1** | **108.90** | **10.6376** | **0.001480** |

## FFMQ – Nonreactivity

|  | **NumDF** | **DenDF** | **F value** | **P value** |
| --- | --- | --- | --- | --- |
| Time | 1 | 113.80 | 2.7533 | 0.09981 |
| Group | 1 | 128.56 | 2.3325 | 0.12916 |
| Origin group | 1 | 123.16 | 1.1462 | 0.28644 |
| FFMQ – Act with awareness | 1 | 854.39 | 1.1945 | 0.27473 |
| **Time * Group** | **1** | **109.46** | **8.8267** | **0.00365** |

## FFMQ – Nonjudging

|  | **NumDF** | **DenDF** | **F value** | **P value** |
| --- | --- | --- | --- | --- |
| **Time** | **1** | **112.26** | **41.8275** | **<.001** |
| **Group** | **1** | **137.86** | **5.5142** | **0.02028** |
| **Origin group** | **1** | **126.92** | **5.5553** | **0.01996** |
| **FFMQ – Act with awareness** | **1** | **889.81** | **69.5388** | **< .001** |
| Time * Group | 1 | 107.49 | 3.4602 | 0.06560 |

## ERQ – Cognitive reappraisal

|  | **NumDF** | **DenDF** | **F value** | **P value** |
| --- | --- | --- | --- | --- |
| Time | 1 | 110.74 | 0.4596 | 0.4992 |
| Group | 1 | 129.77 | 1.9528 | 0.1647 |
| Origin group | 1 | 126.91 | 1.6212 | 0.2053 |
| FFMQ – Act with awareness | 1 | 912.20 | 0.6671 | 0.4143 |
| Time * Group | 1 | 105.72 | 3.6612 | 0.0584 |

## PANAS – Positive affect

|  | **NumDF** | **DenDF** | **F value** | **P value** |
| --- | --- | --- | --- | --- |
| **Time** | **1** | **111.04** | **24.4400** | **<.001** |
| Group | 1 | 128.54 | 0.0126 | 0.91082 |
| Origin group | 1 | 120.09 | 2.5242 | 0.11474 |
| **FFMQ – Act with awareness** | **1** | **896.97** | **28.2791** | **< .001** |
| Time * Group | 1 | 106.08 | 3.7222 | 0.05637 |

## DASS – Depression

|  | **NumDF** | **DenDF** | **F value** | **P value** |
| --- | --- | --- | --- | --- |
| **Time** | **1** | **112.99** | **10.5542** | **0.001527** |
| Group | 1 | 131.12 | 2.2572 | 0.135403 |
| Origin group | 1 | 123.21 | 1.4593 | 0.229354 |
| **FFMQ – Act with awareness** | **1** | **885.21** | **50.7860** | **< .001** |
| **Time * Group** | **1** | **107.93** | **4.0261** | **0.047303** |

## ISI – Insomnia

|  | **NumDF** | **DenDF** | **F value** | **P value** |
| --- | --- | --- | --- | --- |
| **Time** | **1** | **106.99** | **13.7588** | **<.001** |
| Group | 1 | 139.48 | 2.7418 | 0.1000 |
| Origin group | 1 | 120.32 | 0.9366 | 0.3351 |
| **FFMQ – Act with awareness** | **1** | **890.31** | **18.3403** | **< .001** |
| Time * Group | 1 | 103.15 | 2.5256 | 0.1151 |

# Correlation matrix

**Statistically significant correlations are in bold.**

|  | **Frequency of practice** | **Difficulty in practice** |
| --- | --- | --- |
| **FFMQ total score** | ***r* = 0.380**  ***p* = 0.005** | ***r* = -0.450**  ***p* < 0.001** |
| **FFMQ – Observing** | *r* = 0.280  *p* = 0.057 | ***r* = -0.520**  ***p* < 0.001** |
| **FFMQ – Describe** | ***r* = 0.350**  ***p* = 0.047** | *r* = -0.240  *p* = 0.059 |
| **FFMQ – Acting with awareness** | *r* = 0.190  *p* = 0.054 | ***r* = -0.074**  ***p* = 0.021** |
| **FFMQ – Nonjudging** | *r* = 0.160  *p* = 0.074 | ***r* = -0.220**  ***p* = 0.007** |
| **FFMQ – Nonreactivity** | *r* = 0.290  *p* = 0.180 | ***r* = -0.430**  ***p* < 0.001** |
| **ERQ – Cognitive reappraisal** | *r* = 0.180  *p* = 0.130 | ***r* = -0.400**  ***p* = 0.001** |
| **ERQ – Expressive suppression** | *r* = -0.280  *p* = 0.099 | ***r* = 0.330**  ***p* = 0.001** |
| **PANAS – Positive affect** | ***r* = 0.089**  ***p* = 0.019** | ***r* = -0.330**  ***p* < 0.001** |
| **PANAS – Negative affect** | ***r* = -0.380**  ***p* < 0.001** | ***r* = 0.170**  ***p* < 0.001** |
| **DASS total score** | ***r* = -0.290**  ***p* < 0.001** | ***r* = 0.220**  ***p* < 0.001** |
| **DASS – Depression** | ***r* = -0.180**  ***p* < 0.001** | ***r* = 0.260**  ***p* < 0.001** |
| **DASS – Anxiety** | ***r* = -0.260**  ***p* < 0.001** | ***r* = -0.034**  ***p* = 0.009** |
| **DASS – Stress** | ***r* = -0.260**  ***p* =< 0.001** | ***r* = 0.240**  ***p* < 0.001** |
| **RSA total score** | *r* = 0.220  *p* = 0.200 | *r* = -0.058  *p* = 0.740 |
| **RSA – Perception of self** | *r* = 0.250  *p* = 0.150 | *r* = -0.270  *p* = 0.120 |
| **RSA – Planned future** | *r* = 0.160  *p* = 0.350 | *r* = -0.240  *p* = 0.160 |
| **RSA – Social competence** | *r* = -0.041  *p* = 0.810 | *r* = 0.130  *p* = 0.460 |
| **RSA – Family cohesion** | *r* = 0.190  *p* = 0.270 | *r* = 0.026  *p* = 0.880 |
| **RSA – Social resources** | *r* = 0.059  *p* = 0.740 | *r* = 0.036  *p* = 0.840 |
| **RSA – Structured style** | ***r* = 0.360**  ***p* = 0.032** | *r* = 0.083  *p* = 0.640 |
| **ISI – Insomnia** | ***r* = -0.210**  ***p* = 0.002** | ***r* = 0.270**  ***p* < 0.001** |

# Differences in t0

In order to investigate baseline differences between groups, we performed a comparison in t0 between the two groups in all variables of interest. To do this, we ran linear models with group (2-level factor: intervention vs. control) as independent variable and participants’ origin (2-level factor: banking group vs. students and acquaintances) as covariate. Results of group effects are shown in the following table.

NumDF = 1, DenDF = 127.

|  | **F value** | **P value** |
| --- | --- | --- |
| **Habit – Mindfulness practice** | 2.6497 | 0.106 |
| **FFMQ total score** | 0.7010 | 0.404 |
| **FFMQ – Observing** | 2.2970 | 0.132 |
| **FFMQ – Describe** | 1.6877 | 0.196 |
| **FFMQ – Acting with awareness** | **5.3533** | **0.022** |
| **FFMQ – Nonjudging** | 0.0303 | 0.862 |
| **FFMQ – Nonreactivity** | 3.4243 | 0.066 |
| **ERQ – Cognitive reappraisal** | 2.0241 | 0.157 |
| **ERQ – Expressive suppression** | 0.0032 | 0.955 |
| **PANAS – Positive affect** | 1.5801 | 0.211 |
| **PANAS – Negative affect** | 1.622 | 0.205 |
| **DASS total score** | 2.3916 | 0.124 |
| **DASS – Depression** | 0.6597 | 0.418 |
| **DASS – Anxiety** | 2.1081 | 0.149 |
| **DASS – Stress** | 2.6320 | 0.107 |
| **RSA total score** | 0.0590 | 0.808 |
| **RSA – Perception of self** | 0.2702 | 0.604 |
| **RSA – Planned future** | 0.3021 | 0.583 |
| **RSA – Social competence** | 1.3710 | 0.243 |
| **RSA – Family cohesion** | 1.1304 | 0.289 |
| **RSA – Social resources** | 1.2772 | 0.260 |
| **RSA – Structured style** | 2.0636 | 0.153 |
| **ISI – Insomnia** | 1.7127 | 0.193 |

The only statistically significant difference between groups in t0 was found in the FFMQ subscale Acting with awareness (mean intervention group = 11.96; mean control group = 11.02). Given this significant difference, we decided to co-vary this variable in all longitudinal analyses to investigate whether baseline group differences had an influence on the results.
